# Genetic and phenotypic responses of temperature-independent Hessian fly-resistant durum wheat to larval attack during heat stress

**DOI:** 10.1186/s12870-025-06226-1

**Published:** 2025-02-17

**Authors:** Subhashree Subramanyam, Jill A. Nemacheck, Taylor E. Suetsugu, Rachel D. Flynn, Ahmed Faik

**Affiliations:** 1 Crop Production and Pest Control Research Unit, USDA-ARS, West Lafayette, IN 47907 USA; 2 Department of Entomology, Purdue University, West Lafayette, IN 47907 USA; 3 College of Agriculture, Purdue University, West Lafayette, IN 47907 USA; 4 Department of Environmental and Plant Biology, Molecular and Cellular Biology Program, Ohio University, Athens, OH 45701 USA

**Keywords:** *Triticum*, *Mayetiola destructor*, Gall midge, Tetraploid, Global warming, Insect pest, Defense response, Cell wall permeability

## Abstract

**Background:**

Wheat production is increasingly challenged by the devastating damage caused by insect pests. The advent of global warming is further exacerbating this threat. Hessian fly (*Mayetiola destructor*), a dipteran gall midge, is a destructive pest of host wheat (*Triticum aestivum*) having severe economic consequences. Planting wheat cultivars harboring resistance genes is the most effective and economical Hessian fly management strategy. However, heat stress poses a challenge to this strategy, as elevated temperature often breaks down Hessian fly resistance in wheat. Our prior study identified temperature-independent resistant *T. turgidum* (durum wheat) accessions that maintained resistance to Hessian fly when challenged with an increased temperature of 30 °C. In this study, we carried out follow-up characterization of these durum lines to highlight molecular components involved during Hessian fly resistance or susceptibility in wheat following heat stress.

**Results:**

Temperature-independent resistant durum lines were greater than 70% resistant to multiple Hessian fly biotypes at the elevated temperature of 30 °C. At the molecular level, these lines showed increased transcripts of *Hfr-1*, a gene encoding an antinutrient lectin, unlike the heat-triggered susceptible durum wheat. The Hessian fly susceptibility-associated biomarker genes were significantly upregulated in the durum wheat with heat-triggered susceptibility at 30 °C, resembling the gene expression profile observed in susceptible wheat. None of these susceptibility-associated genes were differentially expressed in the temperature-independent resistant wheat. Genes involved in oxidative stress and jasmonic acid pathways did not reveal any specific expression pattern attributed to either heat stress or larval feeding. Neutral red staining revealed limited cell wall permeability in the temperature-independent resistant wheat, unlike the heat-triggered susceptible durum plants that were highly permeable similar to a wheat line susceptible to Hessian fly at 20 °C.

**Conclusions:**

Temperature-independent resistant durum wheat lines provided robust resistance to multiple Hessian fly biotypes at higher temperatures. These lines offer a valuable resource for wheat producers for providing resistance following heat stress.

**Supplementary Information:**

The online version contains supplementary material available at 10.1186/s12870-025-06226-1.

## Background

Insect pests are one of the predominant biotic threats to crop production worldwide and have been associated with severe yield losses and food insecurity. On average, these losses in crop production, due to pests, can range between 10–28% [1]. A boost in insect activity due to climate change further accelerates the rate of crop loss [2]. A yield loss of 10–25% for rice, corn and wheat crops is projected for each 1 °C rise in mean global surface temperature. An estimated annual loss of approximately 213 million tons has been projected for these three cash crops with every 2 °C rise in surface temperature [2]. Wheat (*Triticum aestivum* L.) ranks as the second-most consumed field crop in the world and third in production within the United States (USDA, 2023 https://www.ers.usda.gov/topics/crops/wheat/). Significant global losses in wheat production due to insect pests surmount to a total of around 40 metric megatons annually [2]. The magnitude of these environmental challenges is increasing with global changes in climatic conditions threatening global food security. There is an urgency to identify and characterize molecular factors involved in plant resilience and sensitivity to increased temperature and insect herbivory for developing efficient crop improvement programs.

One of the major destructive pests of wheat is the dipteran gall midge, the Hessian fly (*Mayetiola destructor* [Say]) belonging to the family Cecidomyiidae. This obligate parasite of host wheat causes severe monetary losses impacting wheat growers and producers within the United States and around the world [3–5]. Female Hessian flies lay their eggs on the leaves of young wheat seedlings where they hatch. The newly hatched first instars crawl down to the base of the wheat seedlings to establish feeding sites at the crown. In susceptible plants, the larvae secrete salivary effectors [6] that alter the host plant metabolic pathways [7–9] by suppressing plant defense responses [10] and upregulating susceptibility-associated genes [11–13]. This leads to the formation of a nutritive tissue [14] that provides a nutrient-rich diet [7, 13, 15] and facilitates the diffusion of these nutrients by making the plant cells highly permeable [16]. Establishing wheat host susceptibility makes the plant conducive for the larvae to thrive and complete their development within 28–30 days after egg hatch (DAH). In contrast, avirulent larvae feeding on the resistant wheat accessions, harboring one or more of the documented Hessian fly resistance genes (*H1*-*H36* plus *Hdic*), fail to establish feeding sites. The larvae do not develop beyond the first-instar stage due to starvation and die within 3–5 DAH. The recognition of the insect virulence gene product by the plant Hessian fly resistance (*H*) gene product via gene-for-gene recognition [17] triggers an incompatible interaction (resistant plant) resulting in accumulation of defense and antinutrient proteins [18–21] that disrupt the microvilli within the larval midgut [22].

Planting resistant wheat is the most economical Hessian fly management strategy employed globally. However, this strategy although most effective, comes with challenges including (i) changes in Hessian fly virulence profiles in insect field populations [23, 24]; and (ii) impacts of elevated environmental temperatures on the efficacy of *H* genes in wheat [25–28]. Changes in either or both can ultimately lead to the breakdown of plant resistance. This highlights the urgency to further explore these challenges in-depth to prevent severe wheat yield loss in the event of an outbreak of this insect pest.

Over the past several years, studies have demonstrated that *H* genes in wheat are rendered ineffective or show decreased resistance with rise in environmental temperatures [29]. Several wheat lines with different *H* genes have been shown to lose or have reduced Hessian fly resistance with increasing temperature [25]. Brahmi et al. [26] reported over 50% breakdown of plant resistance in tetraploid durum (*T. turgidum* L.) wheat cultivars to Hessian fly field populations collected from Chaouia-Ouardigha region in North-Central Morocco when exposed to heat stress. A few recent studies have been undertaken to understand the molecular aspects of breakdown of resistance in heat-stressed hexaploid wheat plants harboring the *H13* resistance gene [27, 30, 31]. However, these studies have exclusively focused on heat-stressed plants where resistance is lost. To the best of our knowledge there has been no systematic phenotypic or molecular study directly comparing wheat lines that are able to maintain Hessian fly resistance (temperature-independent resistant) with those that show breakdown of resistance (heat-triggered susceptible) at higher temperatures. Data obtained from such direct comparative studies could offer in-depth information on the mechanisms at play in both heat-triggered susceptible and temperature-independent Hessian fly-resistant wheat lines and further offer an invaluable resource to wheat breeders and producers in utilizing these accessions in downstream programs.

In a recent study, we evaluated the phenotypic response of 254 T*. turgidum* subsp. *durum* (tetraploid) wheat accessions of African origin to biotype L Hessian fly infestation [28]. Of these, 12 durum wheat accessions exhibited a resistance response greater than 70% to biotype L when screened at 20 °C [28]. Interestingly, three of the tetraploid wheat accessions, two originating from Tunisia (CItr 3174 and CItr 6870) and one from Morocco (PI 61862), were also able to maintain 100% resistance (temperature-independent resistance) when subjected to heat stress treatment at 30 °C [28]. In the current study, we have carried out a follow-up study to compare the phenotypic and molecular responses of the temperature-independent resistant durum wheat with that of a heat-triggered susceptible tetraploid wheat that while being resistant at 20 °C is unable to maintain Hessian fly resistance at 30 °C. This study gives us a better understanding of possible mechanisms involved in heat-resilient and heat-compromised Hessian fly resistance in wheat plants.

## Materials and methods

### Insect material

Seven Hessian fly (*Mayetiola destructor*) stocks, biotypes L, B, O, D, C, GP (Great Plains), and *vH13* were used for infestations in the current study. The lab-cultured stocks of these biotypes were maintained in diapause at 4 °C at the USDA-ARS Crop Production and Pest Control Research Unit in West Lafayette, IN, following the methods described by Sosa and Gallun [32].

### Plant material

Five accessions of *Triticum turgidum* subsp. *durum* wheat accessions that were previously phenotyped for their response to biotype L Hessian fly [28] were used in the current study for further characterization of Hessian fly resistance (Table 1). These durum wheat accessions included the lines CItr 6870, CItr 3174, PI 61862, CItr 17647 and CItr 8637. CItr 6870, CItr 3174 and PI 61862 were resistant to Hessian fly biotype L at both 20 °C and 30 °C, while CItr 17647 was resistant at 20 °C but susceptible at 30 °C, and CItr 8637 was susceptible at both 20 °C and 30 °C (Table 1). Seeds for these wheat accessions were procured from the USDA-ARS National Small Grains Collection (Aberdeen, ID).

**Table 1:** *T. turgidum* subsp. *durum* wheat accessions of African origin used in the current study that were demonstrated previously^a ^to show resistance to biotype L Hessian fly

Accession	Origin	Response at 20 °C	Response at 30 °C	Designation
CItr 6870	Tunisia	Resistant	Resistant	Temperature-independent resistant
CItr 3174	Tunisia	Resistant	Resistant	Temperature-independent resistant
PI 61862	Morocco	Resistant	Resistant	Temperature-independent resistant
CItr 17647	Ethiopia	Resistant	Susceptible	Heat-triggered susceptible
CItr 8637^b^	Ethiopia	Susceptible	Susceptible	Susceptible control

^a^Phenotypic response of durum wheat accessions to biotype L infestation as per Nemacheck et al. [28]

^b^Durum wheat used as susceptible control

### Infestation of durum wheat lines with multiple Hessian fly biotypes

The responses of wheat accessions CItr 6870, CItr 3174 and PI 61862 were tested against multiple Hessian fly biotypes B, O, D, C, GP, and *vH13* at two different temperatures, 20 °C and 30 °C. For each interaction at the respective temperature, 2 pots of each line were planted with 10 seeds per 4-inch pot in Pro-Line C/20 Growing Mix (Jolly Gardener Products Inc., Poland Spring, ME). Two additional pots per line were planted to serve as uninfested controls. When the plants reached the 1-leaf stage growing in a Conviron chamber (Controlled Environments Limited, Winnipeg, Manitoba, Canada) set at 20 °C with 16 h:8 h light/dark photoperiod, the pots were covered with vented plastic cups. For ovipositing, 3 mated Hessian fly females were released in the 20 °C treatment pots, while 4 mated females were added to the pots destined for the 30 °C treatment, to compensate for lower infestation levels previously observed in temperature-stressed plants [28]**.** Three days later, approximately 12–24 h prior to egg hatch, 2 infested pots for each interaction and 1 uninfested control pot for each line were moved to another Conviron chamber maintained at 30 °C. Egg hatch the following morning was confirmed by observing sample plants under a dissection microscope for newly-emerged larvae migrating to the base of the plant. Two days after egg hatch (DAH), all pots placed in the 30 °C chamber were returned to the 20 °C chamber. Between 8–11 DAH, plant phenotypes (normal or stunted) were recorded by comparing to the uninfested controls. Infested plants were dissected and the number of dead red (1st instar) or white live (2nd instar) larvae on each plant were counted. Plants showing normal growth and containing dead larvae were counted as resistant; plants with stunted growth and live larvae were counted as susceptible; and plants showing normal growth containing one or more live larvae were counted as tolerant. Representative larval photos were photographed with a DP27 camera mounted on a SZX2 stereomicroscope (Olympus, Center Valley, PA).

### Evaluation of cell wall permeability

To assess epidermal cell wall permeability, plants from the wheat accessions CItr 3174, PI 61862, CItr 17647 and CItr 8637 were evaluated by staining with Neutral Red (NR) as described in Williams et al. [16]. NR staining distinguishes intact plant tissue, which does not absorb stain, from tissue containing cuticular gaps, which absorbs the stain. Darker staining indicates the tissues have absorbed more of the stain and are more permeable, as compared to tissues that appear lighter in color [16]. Plants were grown, infested with Hessian fly biotype L, and subjected to the heat treatment as described above. At 3 DAH, 6–10 plants per treatment were dissected by cutting below the root/crown junction and peeling the first leaf off taking care to avoid injuring the second leaf sheath (location of larvae). The crowns were then stained with 0.1% NR (Sigma-Aldrich, St. Louis, MO) for 10 min followed by thorough rinsing with deionized water. Stained sheaths were scored for intensity of redness on a scale of 0–7 according to Williams et al. [16]. NR-stained uninfested plants and pin-pricked uninfested plants (poked with a 0.2 mm minuten pin) were used as negative and positive controls, respectively. Photomicrographs were taken with a DP27 camera on a SZX2 stereomicroscope (Olympus).

### Transcript profiling of durum wheat lines infested with Hessian fly

To compare the expression profiles of select wheat genes two temperature-independent resistant (CItr 3174 and PI 61862) and one heat-triggered susceptible (CItr 17647) durum wheat lines were chosen. Additionally, a susceptible line (CItr 8637) was also included in this study for comparison. Seeds for these wheat accessions were planted, grown, and infested as described above. Half of the infested and uninfested pots were moved to the 30 °C chamber the evening before egg hatch and returned to the 20 °C chamber 2 DAH, as described above. On 1, 4 and 8 DAH, the second leaf sheaths from both infested and uninfested plants were collected from 4–7 plants per interaction. The tissue samples were frozen immediately in liquid nitrogen and stored in a −80 °C freezer until further use. Collections were repeated for a total of 3 biological replicates. At 11 DAH, the remaining plants (between 3 and 15) were measured from soil level to each leaf blade tip for all treatments. Differences in leaf measurements were evaluated for statistical significance using SAS software version 9.4 (SAS Institute Inc). At 13 DAH, representative photos for each pot of durum wheat accession infested with biotype L Hessian fly at 20 °C and 30 °C were taken. A representative pot containing uninfested control plants was also photographed.

Frozen tissue samples were ground in liquid nitrogen and used for RNA isolation with TRIzol reagent (Invitrogen, Carlsbad, CA) following manufacturer’s instructions. The extracted RNA samples were used as template for cDNA synthesis utilizing the SuperScript IV First-Strand Synthesis system following ezDNase treatment (Invitrogen) according to manufacturer’s protocols. Quantitative real-time reverse transcription polymerase chain reaction (qRT-PCR) was carried out on an Applied Biosystems QuantStudio 6 Pro (Applied Biosystems, Waltham, MA) with primers designed by Primer Express software (Table S1). Briefly, each 10 µl reaction consisted of cDNA (20 ng), 5 µl of 2X PowerUp SYBR master mix (Applied Biosystems) and 0.5 µl each of gene-specific primers (0.5 µM). The following PCR parameters were used: 2 min at 50 °C, 2 min at 95 °C, 40 cycles of 1 s at 95 °C and 30 s at 60 °C. Amplification of a single product was confirmed through a dissociation curve analysis. Each reaction was carried out in triplicate with the ribosomal 18S gene as the endogenous control for normalization. Transcript abundance was quantified by relative standard curve method (ABI User Bulletin 2, ABI PRISM 7700 Sequence Detection System) and significant differences determined by analysis of variance (ANOVA) with SAS software version 9.4 (SAS Institute Inc.) as described previously [33]. Reported fold change values are the ratio of expression in infested tissue to expression in uninfested control samples receiving the same temperature treatments at the same time points.

## Results

### Temperature-independent resistant durum wheat lines maintain resistance to feeding with multiple Hessian fly biotypes

**Fig. 1:**
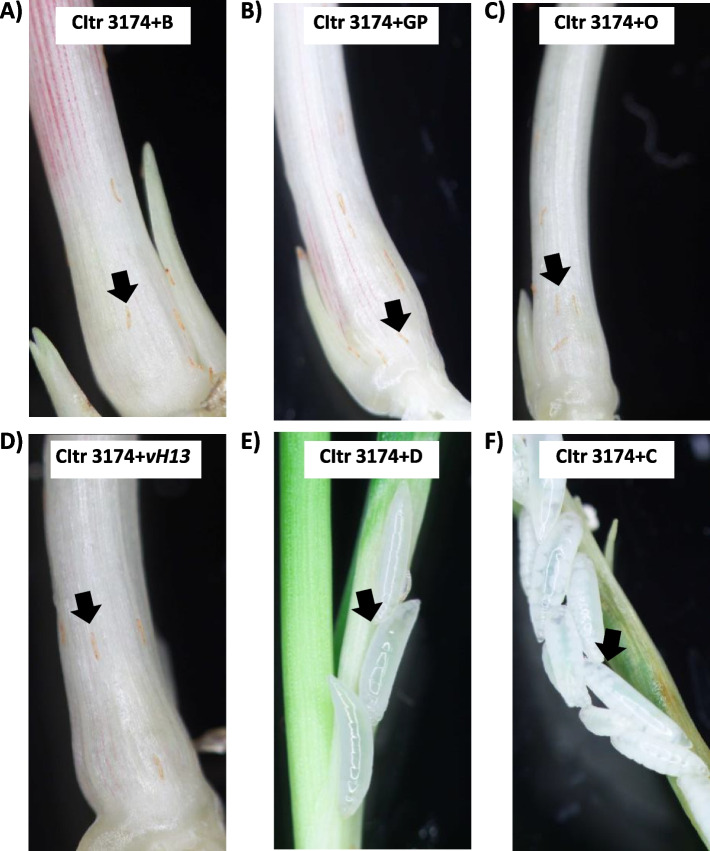
Phenotypic response of durum wheat plants to Hessian fly larval feeding. Representative photographs of CItr 3174 response to multiple Hessian fly biotypes following heat stress at 30 °C between 8–11 DAH (days after egg hatch). The durum wheat line CItr 3174 is temperature-independent resistant to feeding by Hessian fly **A** biotype B; **B** biotype GP (Great Plains); **C** biotype O; and **D** biotype *vH13*. Arrows show dead red 1st-instars on the resistant plants. The durum wheat line CItr 3174 is heat-triggered susceptible to feeding by Hessian fly **E** biotype D; and **F** biotype C. Arrows show live white 2nd or 3rd instars

In our previous study [28], we identified three durum wheat accessions, CItr 6870, CItr 3174 and PI 61862 that were resistant to Hessian fly biotype L infestation even at an increased temperature of 30 °C (temperature-independent resistance). In the current study, we further evaluated and compared the phenotypic response of these three wheat lines to multiple Hessian fly biotypes at both 20 °C and 30 °C. The infestation levels for all biotypes were similar at both temperatures, with averages ranging from 2 to 15 larvae per plant (Tables 2, 3 and 4). Resistance response to multiple larval biotypes was very similar for the durum wheat lines CItr 6870 (Table 2) and CItr 3174 (Table 3), both originating from Tunisia. Both lines showed 80–100% resistance at both temperatures to *vH13* and biotypes GP, B and O with many interactions being 100% resistant. Both the lines were also resistant to biotype D at 20 °C, with CItr 6870 and CItr 3174 exhibiting 88% and 100% resistance, respectively (Tables 2 and 3). In contrast, after heat stress the percentage of resistance to biotype D decreased dramatically in CItr 6870 and CItr 3174 to 44% (Table 2) and 27% (Table 3), respectively. In response to feeding by biotype C, unlike responses observed with other biotypes, these lines showed barely 50% resistance even at the lower temperature of 20 °C which drastically reduced to 0% (CItr 6870) and 24% (CItr 3174) after heat stress (Tables 2 and 3). Representative photomicrographs of CItr 3174 by 8 DAH clearly show resistance response to feeding by biotypes B (Fig. 1A), GP (Fig. 1B), O (Fig. 1C) and *vH13* (Fig. 1D) with all plants harboring only dead red first instars at the crown (larval feeding site) of the wheat seedlings. However, the plants from the durum wheat CItr 3174 showed a susceptible response (plants harboring live 2nd or 3rd instars) to infestation with biotypes D (Fig. 1E) and C (Fig. 1F) clearly indicating that the resistance response breaks down at the higher temperature of 30 °C for these two biotypes. The durum wheat line PI 61862, from Morocco, had different results from the Tunisia lines (Table 4). Although fewer interactions were 100% resistant, the plants remained > 70% resistant to all biotypes used in this study at both temperatures (Table 4).

**Table 2:** Comparative phenotypic response of durum wheat accession CItr 6870 to multiple Hessian fly biotypes at 20** °**C and 30** °**C

	**20 °C**						**30 °C**					
**Biotype**	**# plants**	**Avg. # larvae**	**# R**	**# S**	**# T**	**% R**	**# plants**	**Avg. # larvae**	**# R**	**# S**	**# T**	**% R**
*vH13*	21	9	21	0	0	100	19	10	19	0	0	100
GP	19	12	19	0	0	100	20	10	20	0	0	100
B	17	8	17	0	0	100	13	4	13	0	0	100
O	18	7	15	1	2	83	21	7	20	1	0	95
D	17	5	15	2	0	88	18	7	8	9	1	44
C	15	3	8	7	0	53	16	5	0	13	3	0

# plants: Number of plants phenotyped

Avg # larvae: Average number of larvae per plant

# R: Number of resistant plants showing normal growth and only dead larvae

# S: Number of susceptible plants showing stunted growth and live larvae

# T: Number of tolerant plants showing normal growth with one or more live larvae

% R: Percentage of resistant plants

**Table 3:** Comparative phenotypic response of durum wheat accession CItr 3174 to multiple Hessian fly biotypes at 20** °**C and 30** °**C

	**20 °C**						**30 °C**					
**Biotype**	**# plants**	**Avg. # larvae**	**# R**	**# S**	**# T**	**% R**	**# plants**	**Avg. # larvae**	**# R**	**# S**	**# T**	**% R**
*vH13*	14	4	14	0	0	100	20	4	20	0	0	100
GP	19	9	19	0	0	100	14	3	14	0	0	100
B	20	9	20	0	0	100	18	15	17	1	0	94
O	18	5	17	1	0	94	20	5	20	0	0	100
D	14	4	14	0	0	100	15	9	4	11	0	27
C	20	5	10	4	6	50	21	5	5	10	6	24

# plants: Number of plants phenotyped

Avg # larvae: Average number of larvae per plant

# R: Number of resistant plants showing normal growth and only dead larvae

# S: Number of susceptible plants showing stunted growth and live larvae

# T: Number of tolerant plants showing normal growth with one or more live larvae

% R: Percentage of resistant plants

**Table 4:** Comparative phenotypic response of durum wheat accession PI 61862 to multiple Hessian fly biotypes at 20** °**C and 30** °**C

	**20 °C**						**30 °C**					
**Biotype**	**# plants**	**Avg. # larvae**	**# R**	**# S**	**# T**	**% R**	**# plants**	**Avg. # larvae**	**# R**	**# S**	**# T**	**% R**
*vH13*	10	2	10	0	0	100	11	2	8	3	0	73
GP	13	4	12	1	0	92	16	7	13	3	0	81
B	20	8	17	3	0	85	20	11	14	5	1	70
O	18	7	17	1	0	94	17	7	14	3	0	82
D	18	15	15	3	0	83	18	9	16	1	1	89
C	19	3	19	0	0	100	12	3	10	2	0	83

# plants: Number of plants phenotyped

Avg # larvae: Average number of larvae per plant

# R: Number of resistant plants showing normal growth and only dead larvae

# S: Number of susceptible plants showing stunted growth and live larvae

# T: Number of tolerant plants showing normal growth with one or more live larvae

% R: Percentage of resistant plants

### Temperature-independent resistant durum wheat shows normal leaf and plant growth

**Fig. 2:**
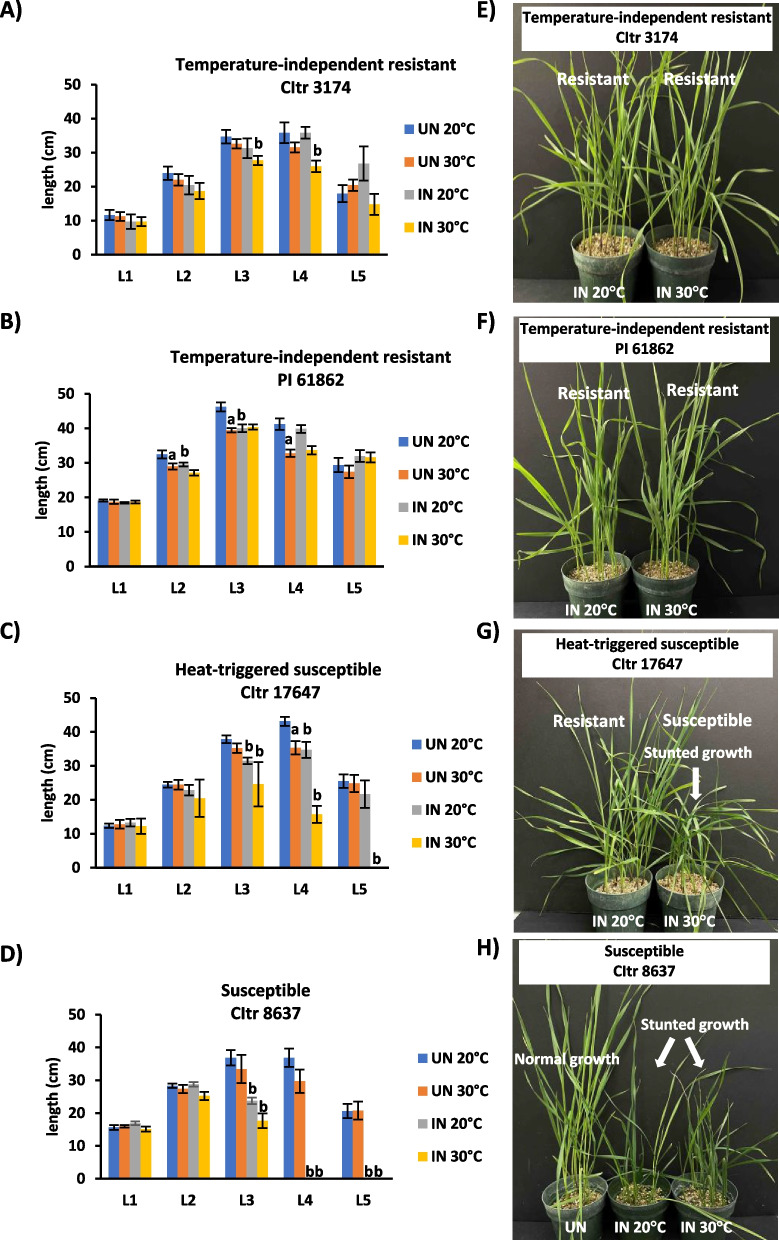
Leaf and plant growth in durum wheat at 20 °C and 30 °C. Length measurements for main stem leaves 1 to 5 (L1-L5) were taken on 11 DAH for two temperature-independent resistant wheat accessions **A** CItr 3174; **B** PI 61862, one heat-triggered susceptible wheat accession **C** CItr 17647 and the susceptible wheat accession **D** CItr 8637 at 20 °C and 30 °C. Means of leaf lengths of uninfested (UN) plants that were significantly shorter (*p* < 0.05) following the 30 °C treatment compared to the same leaves on plants kept at 20 °C are indicated with an “a” above the bar. Means of leaf lengths of Hessian fly biotype L-infested plants (IN) that were significantly shorter (*p* < 0.05) than the same leaves on uninfested plants receiving the same temperature treatment are indicated with a “b” above the bar. Representative photos of pots showing plant growth taken 13 DAH for biotype-L infested durum wheat accessions at 20 °C and 30 °C are depicted in E–H. The two temperature-independent resistant durum wheat lines **E** CItr 3174 and **F** PI 61862 are resistant with normal plant growth at both temperatures. The heat-triggered susceptible wheat line **G** CItr 17647 is susceptible with stunted plant growth at 30 °C, as compared to normal plant growth at 20 °C. The biotype L-infested susceptible durum wheat line **H** CItr 8637 shows stunted plant growth at both temperature treatments compared to uninfested plants that show normal growth

The treatment of three days at 30 °C had some small but significant effects (*p* < 0.05) on leaf growth for all durum wheat accessions (Fig. 2A-D). For both, the temperature-independent resistant wheat line (CItr 3174) (Fig. 2A) and the susceptible wheat line (CItr 8637) (Fig. 2D), all leaves for uninfested plants grew at similar rates regardless of temperature treatment. However, in the other temperature-independent resistant (PI 61862) durum wheat, leaves 2–4 from the uninfested plants were mildly stunted following 30 °C treatment (*p* = 0.0275, 0.0003 and 0.0004, respectively) (Fig. 2B). Similarly, leaf 4 on the heat-triggered susceptible CItr 17647 (*p* = 0.0022) uninfested wheat plants was also mildly stunted (Fig. 2C). In both these lines, growth in uninfested plants returned to normal in leaf 5 (Fig. 2B and C). For both temperature-independent resistant lines, infestation caused some mild stunting of younger leaves (CItr 3174 leaves 3 and 4, *p* = 0.0192 and 0.0218; PI 61862 leaves 2 and 3, *p* = 0.0188 and 0.002), but growth returned to normal for leaf 5 (Fig. 2A and B). At 20 °C, the heat-triggered susceptible wheat line grew similarly compared to the temperature-independent resistant lines, with mild stunting of leaves 3 and 4 (*p* = 0.017 and 0.028). However, when resistance was lost following heat stress, plant growth was arrested early as evident by the degree of stunting in leaf 4 (*p* = 0.0004) and complete lack of a leaf 5 for CItr 17647 at 30 °C (Fig. 2C). At both temperature treatments, the susceptible line, CItr 8637, had a stunted leaf 3 (*p* < 0.0001 at 20 °C, *p* = 0.0017 at 30 °C) and complete lack of leaves 4 and 5 (Fig. 2D). The whole plant photographs taken on 13 DAH of biotype L-infested durum wheat accessions clearly showed that increased temperature (30 °C) did not have any noticeable effect on plant growth for the temperature-independent resistant (CItr 3174 and PI 61862) lines, exhibiting normal growth similar to the plants growing at 20 °C (Fig. 2E and F). In contrast, the heat-triggered susceptible wheat line (CItr 17647) when subjected to heat stress showed stunted growth unlike the resistant plant at 20 °C, but resembling the response observed in the susceptible wheat (CItr 8637) accession (Fig. 2G and H). The susceptible wheat accession showed similar responses (stunting) at both temperatures as compared to the uninfested control plants (Fig. 2H).

### Decreased epidermal cell wall permeability in temperature-independent resistant durum wheat

**Fig. 3:**
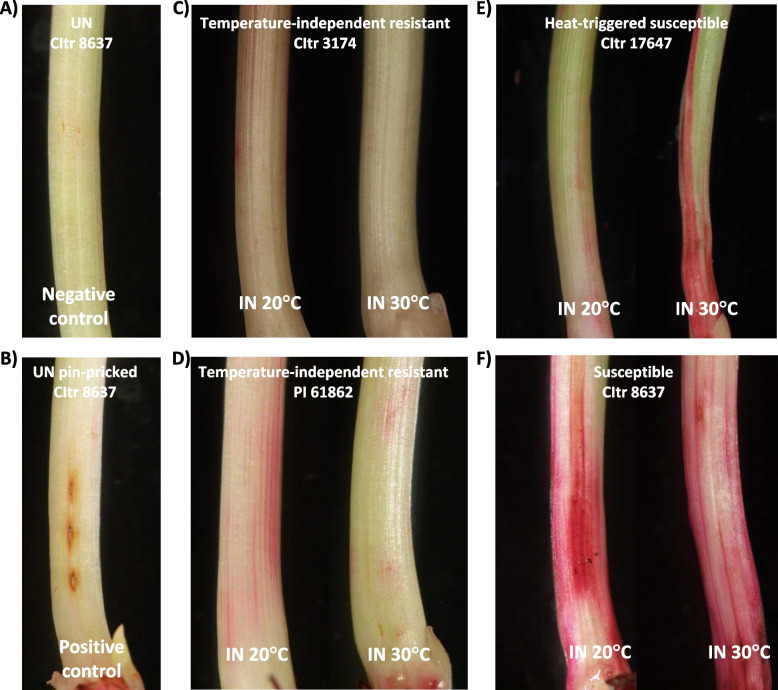
Changes in epidermal cell wall permeability in durum wheat accessions at 20 °C and 30 °C. Biotype L-infested plants from durum wheat accessions, subjected to 20 °C and 30 °C treatments were stained with Neutral Red (NR) and scored at 3 DAH (days after egg hatch). **A** Representative uninfested (UN) CItr 8637 plant included as negative control. **B** Representative uninfested CItr 8637 plant was pin-pricked and used as positive control to distinguish staining caused by larval feeding from that caused by physical damage. Biotype L-infested (IN) and NR-stained **C** CItr 3174 at 20 °C and 30 °C; **D** PI 61862 at 20 °C and 30 °C; **E** CItr 17647 at 20 °C and 30 °C; **F** CItr 8637 at 20 °C and 30 °C

To compare epidermal cell wall permeability in temperature-independent resistant and heat-triggered susceptible durum wheat lines, the Hessian fly biotype L-infested plants exposed to 20 °C and 30 °C were stained with Neutral Red (NR) and scored as described previously [16]. Representative photomicrographs for the negative (uninfested) and positive (pin-pricked and uninfested) controls for NR staining are shown in Fig. 3A and B, respectively. The plants from the temperature-independent resistant wheat accession (CItr 3174) had very little staining, appearing as lines in the crown (larval feeding site), with 67% of the plants scoring 0, and looking like uninfested controls (Fig. 3C). At both temperature treatments, scores ranged from 0–2 for each CItr 3174 plant, averaging 0.7 ± 0.4 for the 20 °C plants and 0.5 ± 0.3 for the 30 °C plants (Table 5). At 20 °C, the temperature-independent resistant wheat plants (PI 61862) were all resistant and scores ranged 1–3 (average 2.1 ± 0.2), typical of other resistant wheat plants, but higher than CItr 3174 (Fig. 3D). Interestingly, at 30 °C, both resistant (harboring only dead larvae) and susceptible plants (harboring dead reds plus live larvae) were found in the heat stressed PI 61862 plants designated as PI 61862 R and PI 61862 S, respectively (Table 5). This is not surprising as PI 61862 was not 100% resistant to any of the biotypes tested when subjected to heat stress (Table 4). At 30 °C the 7 resistant plants (PI 61862 R) scored 1–2, averaging 1.4 ± 0.2 (Fig. 3D), while the 3 susceptible plants (PI 61862 S) scored 4–6, averaging 5.3 ± 0.7 (Table 5). Two of these susceptible plants (PI 61862 S) had only a single live larva along with > 10 dead reds, but that single larva was enough to cause typical susceptibility scores. The heat-triggered susceptible wheat line (CItr 17647) had scores of 1–3 (average 1.9 ± 0.3) for resistant plants at 20 °C and scores ranging 1–6 (average 4.6 ± 0.6) for the susceptible plants at 30 °C (Table 5) with NR staining appearing around the entire length of the crown (Fig. 3E). One of these plants had a score of 1, like a resistant plant; however, 6 of the 9 plants had scores of 5 or 6, typical of susceptible plants (Table 5). The susceptible wheat line (CItr 8637) was highly permeable, as expected, with scores of 4–7 at both temperature treatments, averaging 5.7 ± 0.5 at 20 °C and 6.0 ± 0.4 at 30 °C (Table 5) with NR stain appearing as a blush covering a large area around the entire length of the crown tissue (Fig. 3F).

**Table 5:** Assessment of cell permeability^a ^in Hessian fly biotype L-infested durum wheat plants subjected to 20** °**C and 30** °**C

	**20 °C**				**30 °C**			
**Accession**	**# plants**	**Avg.** **# live larvae**	**Avg.** **# dead larvae**	**Avg. NR** **score ± SE**	**# plants**	**Avg.** **# live larvae**	**Avg.** **# dead larvae**	**Avg. NR** **score ± SE**
CItr 3174	6	0.0	9.5	0.7 ± 0.4	6	0.0	4.2	0.5 ± 0.3
PI 61862 R^b^	10	0.0	16.7	2.1 ± 0.2	7	0.0	17.0	1.4 ± 0.2
PI 61862 S^c^	0	-	-	-	3	3.3	17.3	5.3 ± 0.7
CItr 17647	9	0.0	16.7	1.9 ± 0.3	9	37.6	2.9	4.6 ± 0.6
CItr 8637	6	11.3	0.2	5.7 ± 0.5	6	19.2	0.0	6.0 ± 0.4

^a^Wheat plants were dissected on 3 DAH (days after egg hatch) to expose the feeding sites, stained with Neutral Red (NR), and the intensity of the red stain was scored on a scale of 0–7, as described previously in Williams et al. [16]

^b^PI 61862 resistant plants at 30 °C harboring only dead larvae

^c^PI 61862 susceptible plants at 30 °C harboring dead and live larvae

# plants: Total number of plants per wheat accession stained with NR

Avg. # live larvae: Average number of live larvae per plant from each wheat accession

Avg. # dead larvae: Average number of dead larvae per plant for each wheat accession

Avg. NR score ± SE: Average NR score and standard error

### Differential expression of Hessian fly-responsive defense response biomarker genes in durum wheat

**Fig. 4:**
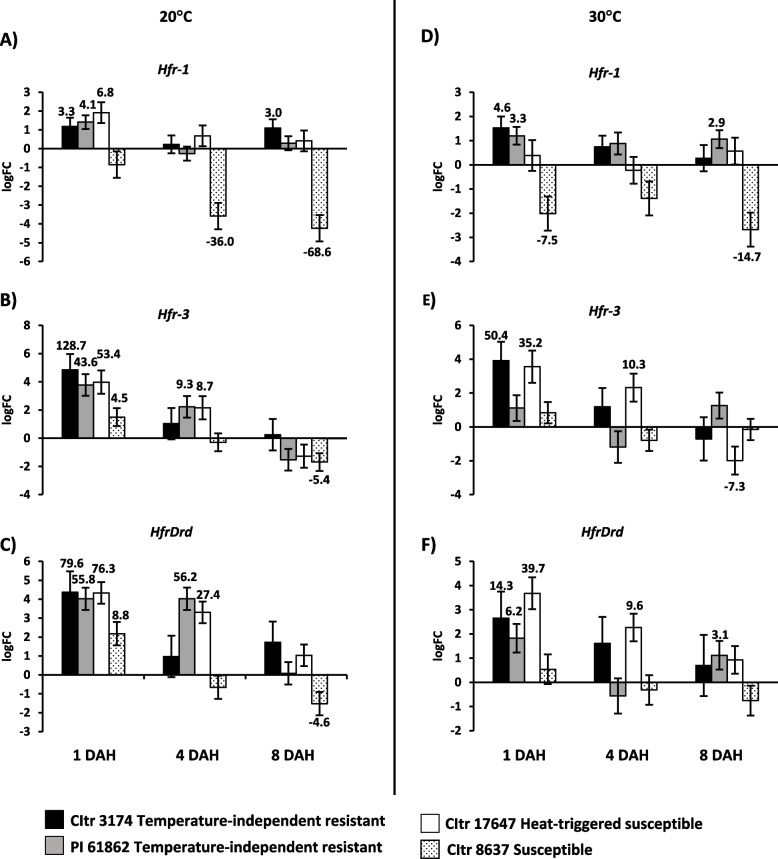
Expression of Hessian fly-responsive defense response biomarker genes in durum wheat seedlings in response to larval feeding at 20 °C and 30 °C. Transcript levels for *Hfr-1* (Hessian fly response-1), *Hfr-3* (Hessian fly responsive-3), and *HfrDrd* (Hessian fly-responsive dirigent-like protein) are shown as logarithmic fold change (logFC) of infested vs uninfested plants for each wheat line at 20 °C (**A**-**C**) and 30 °C (**D**-**F**). The gene expression data were assessed at three time-points, 1, 4, and 8 DAH (days after egg-hatch). Only significant fold changes (*p* < 0.05) are indicated above (upregulated) or below (downregulated) the bar. The temperature-independent resistant wheat lines CItr 3174 and PI 61862 are indicated as black and grey bars, respectively. The heat-triggered susceptible wheat line CItr 17647 is shown as a white bar. The susceptible wheat line CItr 8637 is shown as a dotted bar

The expression of select defense-associated genes that serve as biomarkers during wheat resistance (incompatible interaction) to Hessian fly infestation was profiled in temperature-independent resistant and heat-triggered susceptible durum wheat lines over a time course at both temperatures (Fig. 4). Genes associated with resistance to Hessian fly, such as *Hfr-1* (Hessian fly response gene-1), *Hfr-3* (Hessian fly responsive-3) and *HfrDrd* (Hessian fly-responsive disease-resistance dirigent-like protein) showed expected expression profiles in all 3 Hessian fly resistant lines, CItr 3174, PI 61862, and CItr 17647 at 20 °C, being induced significantly at higher levels compared to the susceptible wheat (CItr 8637) that showed much less (*HfrDrd* and *Hfr-3*) to no (*Hfr-1*) increased transcript levels (Fig. 4A-C). Heat stress at 30 °C caused some changes in the expression of these genes (Fig. 4D-F). In both the temperature-independent resistant durum wheat lines (CItr 3174 and PI 61862), *Hfr-1* expression was very similar between 20 °C and 30 °C, but this gene was no longer induced in the heat-triggered susceptible durum wheat (CItr 17647) line at 30 °C (Fig. 4D). In contrast, *Hfr-3* (35.2-fold; *p* = 0.002) and *HfrDrd* (39.7-fold; *p* < 0.001) were still induced in the heat-triggered susceptible line after heat stress at 1 DAH and levels remained high through 4 DAH (*Hfr-3* 10.3-fold; *p* = 0.012 and *HfrDrd* 9.6-fold; *p* = 0.001) despite resistance being lost in this line. As expected, all three Hessian fly-responsive defense genes were either mostly not expressed or significantly down-regulated (*p* < 0.05) in the susceptible durum wheat line CItr 8637 over the time course at both temperature treatments (Fig. 4A-F).

### Differential expression of Hessian fly-responsive susceptibility-associated biomarker genes in heat-triggered susceptible durum wheat

**Fig. 5:**
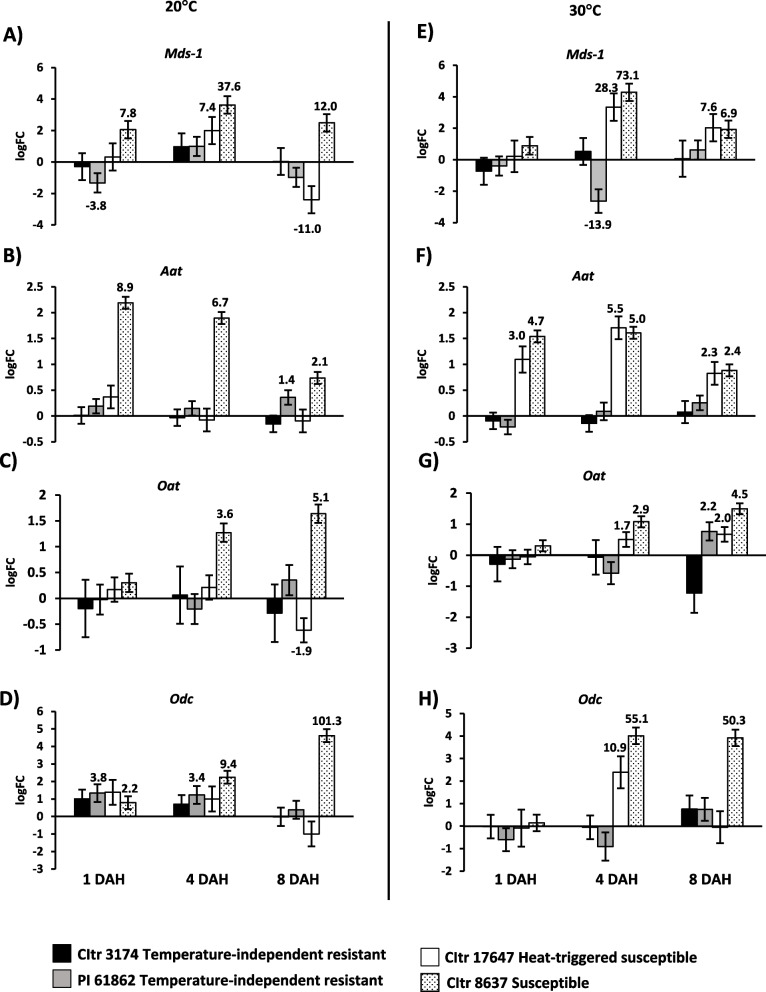
Expression of Hessian fly-responsive susceptibility-associated biomarker genes in durum wheat seedlings in response to larval feeding at 20 °C and 30 °C. Transcript levels for *Mds-1 (Mayetiola destructor* susceptibility-1), *Aat* (Transmembrane amino acid transporter), Oat (Ornithine aminotransferase), and *Odc* (Ornithine decarboxylase) are shown as logarithmic fold change (logFC) of infested vs uninfested plants for each wheat line at 20 °C (**A**-**D**) and 30 °C (**E**-**H**). The gene expression data were assessed at three time-points, 1, 4, and 8 DAH (days after egg-hatch). Only significant fold changes ( *p* < 0.05) are indicated above (upregulated) or below (downregulated) the bar. The temperature-independent resistant wheat lines CItr 3174 and PI 61862 are indicated as black and grey bars, respectively. The heat-triggered susceptible line CItr 17647 is shown as a white bar. The susceptible wheat line CItr 8637 is shown as a dotted bar

Marker genes induced during compatible wheat-Hessian fly interactions (susceptibility), such as *Mds-1* (*Mayetiola destructor* susceptibility 1), *Aat* (Transmembrane amino acid transporter), *Oat* (Ornithine aminotransferase), and *Odc* (Ornithine decarboxylase) were profiled in the temperature-independent resistant and heat-triggered susceptible durum wheat lines over a time course at 20 °C and 30 °C (Fig. 5). As expected, the transcripts for all four susceptibility-associated genes were significantly (*p* = 0.001) induced to higher levels in the susceptible wheat (CItr 8637) at most time points and both temperatures as compared to the uninfested controls (Fig. 5A-H). While not all four genes (except *Mds-1* at 4 DAH) were induced at higher levels at 20 °C in the heat-triggered susceptible durum wheat CItr 17467 (Fig. 5A-D), they were all significantly (*p* < 0.001) induced to higher levels following heat stress treatment at 30 °C, resembling the expression profile observed in susceptible wheat CItr 8637 (Fig. 5E-H). Except for *Aat* (Fig. 5F) that showed a significant increase in transcripts at 1 DAH (threefold; *p* < 0.001), the expression of *Mds-1*, *Oat*, and *Odc* was significantly induced only by 4 DAH in the heat-triggered susceptible wheat CItr 17647, and susceptible wheat CItr 8637 (Fig. 5E, G and H). At 4 DAH, the transcript levels for *Mds-1* and *Odc* showed significantly higher fold changes (Fig. 5E and H), as compared to the lower fold changes observed for *Aat* and *Oat* in the heat-triggered susceptible wheat (CItr 17647) following heat stress (Fig. 5F and G). Except for a slight increase in transcripts of *Odc* in the temperature-independent resistant wheat at 20 °C by 1 DAH (3.8-fold; *p* = 0.019) and 4 DAH (3.4-fold; *p* = 0.028), none of the other susceptibility-associated genes were differentially expressed in either of the two temperature-independent resistant PI 61862 wheat lines at both temperatures and all time points (Fig. 5).

### Downregulation of cell wall-associated genes in heat-triggered susceptible durum wheat

**Fig. 6:**
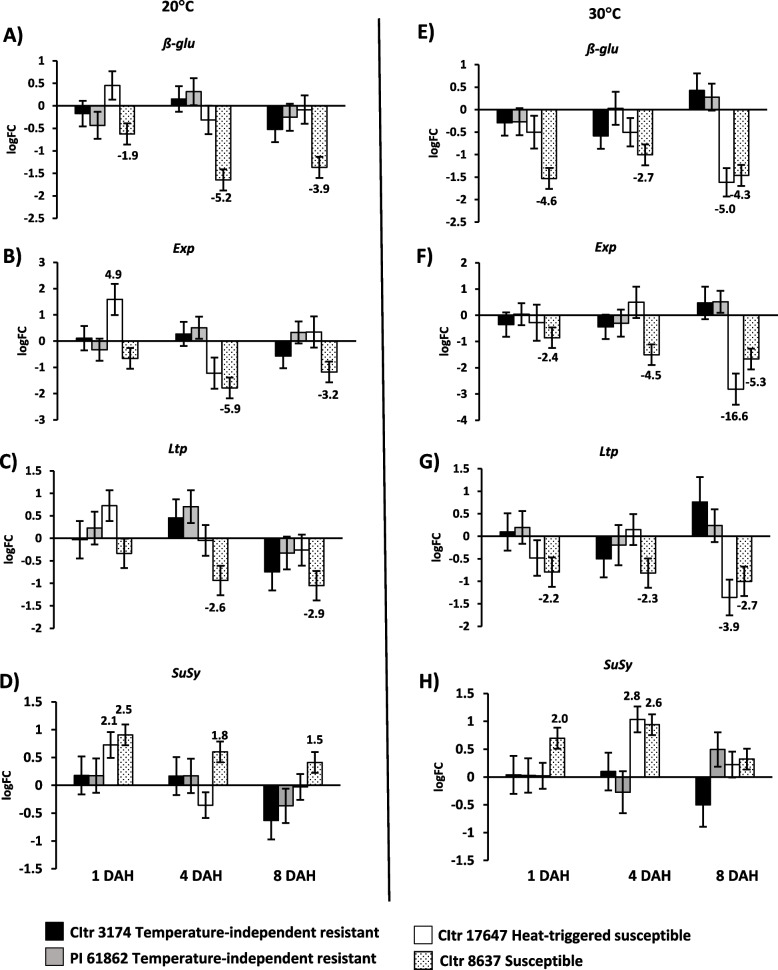
Expression of cell wall-associated genes in durum wheat seedlings in response to larval feeding at 20 °C and 30 °C. Transcript levels for *ß-glu* (Beta-glucosidase), *Exp* (Expansin), *Ltp* (Lipid transfer protein), and *SuSy* (Sucrose synthase) are shown as logarithmic fold change (logFC) of infested vs uninfested controls for each wheat line at 20 °C (**A**-**D**) and 30 °C (**E**–**H**). The gene expression data were assessed at three time-points, 1, 4, and 8 DAH (days after egg-hatch). Only significant fold changes (*p* < 0.05) are indicated above (upregulated) or below (downregulated) the bar. The temperature-independent resistant wheat lines CItr 3174 and PI 61862 are indicated as black and grey bars, respectively. The heat-triggered susceptible line CItr 17647 is shown as a white bar. The susceptible wheat line CItr 8637 is shown as a dotted bar

Since differential cell wall permeability changes were observed between the temperature-independent resistant and heat-triggered susceptible durum lines, we wanted to better understand how larval feeding affects expression of plant cell wall-associated genes in these wheat lines. We included key biomarker genes identified in susceptible hexaploid wheat in response to Hessian fly infestation [9] in this study. The transcripts for cell wall-associated genes *ß-glu* (Beta-glucosidase), *Exp* (Expansin), and *Ltp* (Lipid transfer protein) were significantly (*p* < 0.05) suppressed in the susceptible wheat (CItr 8637) at both 20 °C (Fig. 6A-C) and 30 °C (Fig. 6E-G) in most time points. These genes were also significantly suppressed in the heat-triggered susceptible wheat (CItr 17647), but not until 8 DAH and only at 30 °C with *ß-glu* (Fig. 6E), *Exp* (Fig. 6F), and *Ltp* (Fig. 6G) down-regulated fivefold (*p* < 0.001), 16.6-fold (*p* < 0.001), and 3.9-fold (*p* = 0.003), respectively as compared to the uninfested controls. None of these three genes were significantly expressed in either of the two temperature-independent resistant wheat lines (CItr 3174 and PI 61862) at any time point or temperature (Fig. 6A-C and E–G). The cell wall gene encoding Sucrose Synthase (*SuSy*) showed a slight but significant increase in transcript levels at 1 DAH at 20 °C (2.1-fold; *p* = 0.006) and 4 DAH at 30 °C (2.8-fold; *p* < 0.001) in the heat-triggered susceptible durum wheat (CItr 17647), resembling the profile observed in susceptible wheat (Fig. 6D and H). None of the temperature-independent resistant wheat lines showed differential expression of transcripts for *SuSy* at either of the temperatures (Fig. 6D and H).

### Expression of oxidative stress pathway genes in durum wheat

**Fig. 7:**
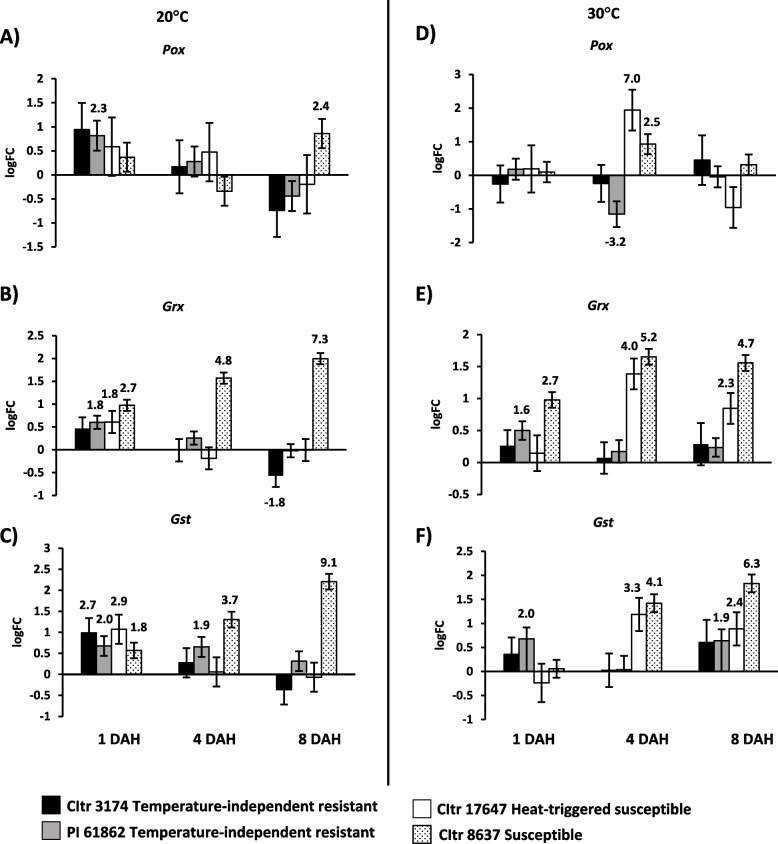
Expression of oxidative stress pathway genes in durum wheat seedlings in response to larval feeding at 20 °C and 30 °C. Transcript levels for *Pox* (Class III peroxidase), *Grx* (Glutaredoxin), and *Gst* (Glutathione S-transferase) are shown as logarithmic fold change (logFC) of infested vs uninfested controls for each wheat line at 20 °C (**A**-**C**) and 30 °C (**D**-**F**). The gene expression data were assessed at three time-points, 1, 4, and 8 DAH (days after egg-hatch). Only significant fold changes (*p* < 0.05) are indicated above (upregulated) or below (downregulated) the bar. The temperature-independent resistant wheat lines CItr 3174 and PI 61862 are indicated as black and grey bars, respectively. The heat-triggered susceptible line CItr 17647 is shown as a white bar. The susceptible wheat line CItr 8637 is shown as a dotted bar

To determine if reactive oxygen species (ROS) are involved in the durum wheat resistance or susceptibility to Hessian fly at specific temperature treatments, transcripts for genes involved in oxidative burst, including *Pox* (Class III Peroxidase), *Grx* (Glutaredoxin) and *Gst* (Glutathione *S*-transferase) were profiled (Fig. 7). Transcripts for all three genes significantly (*p* < 0.05) increased in the susceptible wheat in at least one or more time-points at 20 °C (Fig. 7A-C) and 30 °C (Fig. 7D-F). Following heat stress, the heat-triggered susceptible wheat line (CItr 17647) showed significantly increased transcripts for *Pox* (sevenfold; *p* = 0.005); *Grx* (fourfold; *p* < 0.001) and *Gst* (3.3-fold; *p* < 0.001) at 4 DAH but stayed up at 8 DAH only for *Grx* (2.3-fold; *p* = 0.003) and *Gst* (2.4-fold; *p* = 0.020) transcripts resembling the expression profile observed for the susceptible wheat line CItr 8637 (Fig. 7D-F). Interestingly, significantly increased transcript levels were also observed for *Pox*, *Grx* and *Gst* in one or both temperature-independent resistant wheat lines (CItr 3174 and PI 61862) at one or more time points at 20 °C (Fig. 7A-C). Upregulated transcripts for *Grx* (1.6-fold; *p* = 0.003) and *Gst* (twofold; *p* = 0.011) were also observed in one of the temperature-independent resistant wheat lines (PI 61862) following heat stress (Fig. 7E and F).

### Expression of genes involved in jasmonic acid (JA) biosynthesis in durum wheat

**Fig. 8:**
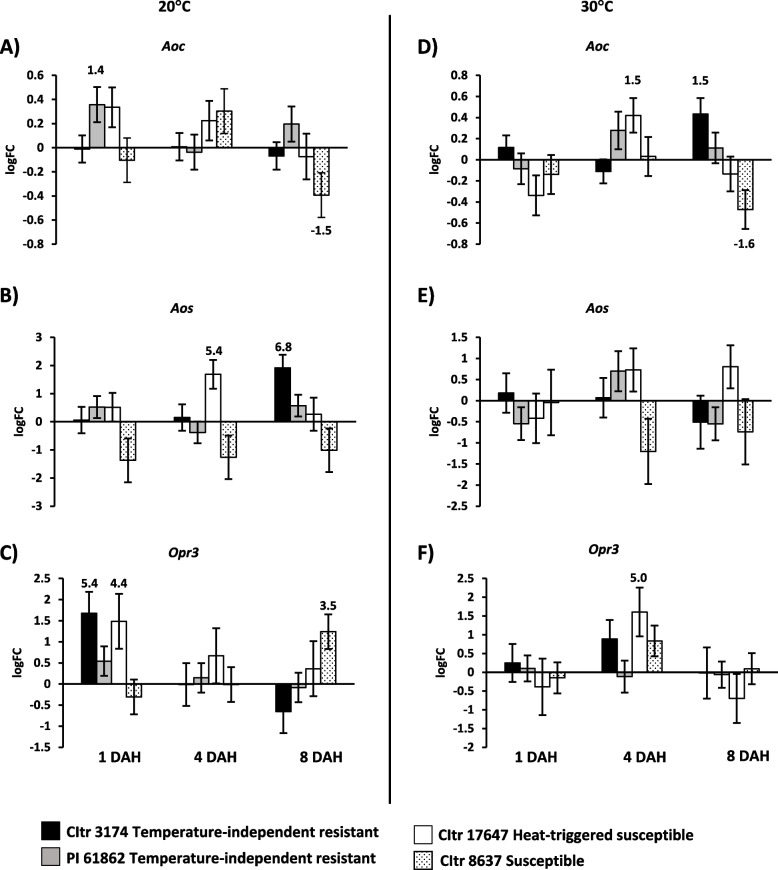
Expression of jasmonic acid (JA) pathway genes in durum wheat seedlings in response to larval feeding at 20 °C and 30 °C. Transcript levels for *Aoc* (Allene oxide cyclase), *Aos* (Allene oxide synthase), and *Opr3* (12-oxophytodienoate reductase) are shown as logarithmic fold change (logFC) of infested vs uninfested plants for each wheat line at 20 °C (**A**-**C)** and 30 °C (**D**-**F**). The gene expression data were taken at three time-points, 1, 4, and 8 DAH (days after egg-hatch). Only significant fold changes (*p* < 0.05) are indicated above (upregulated) or below (downregulated) the bar. The temperature-independent resistant wheat lines CItr 3174 and PI 61862 are indicated as black and grey bars, respectively. The heat-triggered susceptible line CItr 17647 is shown as a white bar. The susceptible wheat line CItr 8637 is shown as a dotted bar

Since jasmonic acid (JA) pathway genes are implicated in wheat-Hessian fly interactions under heat stress [27] we investigated transcripts of key genes in JA biosynthesis that included *Aoc* (Allene oxide cyclase), *Aos* (Allene oxide synthase), and *Opr3* (12-oxo-phytodienoate reductase) in the temperature-independent resistant and heat-triggered susceptible durum wheat lines at 20 °C and 30 °C over time. The expression pattern for these genes did not reveal a specific pattern with transcripts differentially expressing at different time points/temperatures and genotypes (Fig. 8). At 20 °C by 1 DAH significant levels of transcripts for *Aoc* (1.4-fold; *p* = 0.026) were accumulated in PI 61862 (Fig. 8A), however, *Opr3* transcripts showed increased levels in CItr 3154 (5.4-fold; *p* = 0.004) and CItr 17647 (4.4-fold; *p* = 0.036) wheat lines (Fig. 8C). In contrast, transcripts for *Aos* were only up by 8 DAH in the temperature-independent resistant wheat line CItr 3154 (Fig. 8B). After the 30 °C heat stress treatment, while *Aos* transcript levels were not significantly changed in any of the genotypes, *Opr3* transcripts were significantly upregulated only in heat-triggered susceptible wheat (CItr 17467) at 4 DAH (fivefold; *p* = 0.025) similar to the expression pattern observed for *Aoc* transcripts (1.5-fold; *p* = 0.021) at 4 DAH. The transcripts for *Aoc* also increased 1.5-fold (*p* = 0.011) by 8 DAH in the temperature-independent resistant wheat line (CItr 3154) following heat stress (Fig. 8D). Like the other durum wheat lines, no specific gene expression profile was observed in the susceptible wheat (CItr 8637) for both temperatures (Fig. 8). While transcripts for *Aoc* (Fig. 8A and D) were significantly downregulated by 8 DAH at 20 °C (1.5-fold; *p* = 0.047) and 30 °C (1.6-fold; *p* = 0.020), *Opr3* transcripts were significantly upregulated by 8 DAH (3.5-fold; *p* = 0.008) at 20 °C (Fig. 8C).

## Discussion

Elevated temperatures (25–30 °C) render Hessian fly resistance genes ineffective or result in reduced resistance in wheat to larval attack [25–28, 31, 34, 35]. In recent years, a few studies have been undertaken to identify molecular factors involved in heat-stressed wheat and resistance to Hessian fly [27, 30, 31]. However, much of this work has been focused only on the hexaploid wheat ‘Molly’ harboring the *H13* resistance gene, a line that is resistant to Hessian fly at 20 °C but loses resistance at higher temperature (30 °C). Further, these studies characterize factors involved in loss of resistance at higher temperature, leaving a big gap in our understanding of factors in wheat lines that can maintain resistance to the insect pest despite being exposed to elevated temperatures [28]. In a recent study, we phenotyped 254 tetraploid durum wheat (*T. turgidum*) lines of African origin against Hessian fly biotype L and identified accessions that were: (i) temperature-independent resistant (plants showing Hessian fly resistance at 20 °C and 30 °C, and (ii) heat-triggered susceptible (plants showing Hessian fly resistance at 20 °C but not at 30 °C). Here we discuss the results of a follow-up study to compare the heat-resilient and -compromised Hessian fly-responsive durum wheat lines at the phenotypic and molecular levels to better understand the wheat-Hessian fly interactions exposed to lower and higher temperatures.

The three temperature-independent resistant durum wheat lines (CItr 6870, CItr 3174 and PI 61862) showed resistance to multiple Hessian fly biotypes, in addition to the biotype L reported previously [28]. While the wheat line PI 61862 from Morocco, remained > 70% resistant to all the 6 Hessian fly biotypes at both temperatures (Table 4) the other two durum wheat lines CItr 6870 and CItr 3174, both originating from Tunisia, showed 80–100% resistance to 4 biotypes (*vH13*, GP, B, O), but were sensitive to biotypes C and D at 30 °C (Tables 2 and 3). The classical host wheat-Hessian fly interaction involves the recognition of the insect virulence gene product by the plant resistance gene product resulting in larval antibiosis within 5 DAH and the wheat plant being resistant [17]. Our results clearly show the impact of heat stress on either this recognition in CItr 6870 and CItr 3174 at higher temperatures when fed by biotypes C and D, or on the blocking of some signaling pathways downstream of this recognition that do not induce the same defense response pathways.

Plants respond to heat stress at multiple levels to adapt, survive and develop in stressed environments [36]. This additional environmental stress may also affect the ability of plants to defend against biotic stressors. While Hessian fly infestation did cause some mild stunting of younger leaves (2- 4) in the temperature-independent resistant durum wheat lines (CItr 3174 and PI 61862), there was no growth penalty to the plant as leaf 5 showed normal growth despite being subjected to heat stress at 30 °C. The Hessian fly-infested plants from these accessions showed comparable plant growth at both temperatures (Fig. 2E and F). In contrast, unlike at 20 °C, loss of resistance following exposure to higher temperature in the heat-triggered susceptible wheat (CItr 17647) resulted in stunted leaves and arrested plant growth (Fig. 2G) resembling the stunted growth observed in the Hessian fly-infested susceptible line (CItr 8637) at both temperatures as compared to uninfested control plants showing normal growth (Fig. 2H). The mild stunting observed in the temperature-independent resistant durum wheat lines could be an adaptive feature to cope with the stress. In contrast, in the durum wheat line with heat-triggered susceptibility, a possible loss of *H* gene function or loss of the corresponding avirulence effector from Hessian fly at higher temperatures results in arrested plant growth resembling the response observed in the susceptible wheat.

Insect herbivory triggers expression of defense response genes in plants as a mechanism to cope with the stress and prevent or reduce the damage [37–39]. Hessian fly larval attack triggers elevated expression of defense response genes including *Hfr-1*, *Hfr-3* and *HfrDrd* in resistant hexaploid wheat genotypes and nonhost rice and *Brachypodium* plants [19, 21, 33, 40, 41]. These genes serve as biomarkers involved in wheat defense against Hessian fly. Transcript analysis of these genes revealed significant upregulation in both the temperature-independent resistant (CItr 3174 and PI 61862) and heat-triggered susceptible (CItr 17647) durum wheat lines following Hessian fly larval attack as early as 1 DAH at 20 °C (Fig. 4A-C). These results were expected as all three lines previously showed > 80% resistance to biotype L Hessian fly at this temperature [28]. However, at elevated temperature treatment of 30 °C, *Hfr-1* transcripts were significantly upregulated only in the temperature-independent resistant wheat lines but not in the heat-triggered susceptible wheat as compared to the uninfested controls (Fig. 4D). Unlike transcripts for *Hfr-1*, the mRNA levels for *HfrDrd* and *Hfr-3* were found to be upregulated in both types of wheat lines at 30 °C at both early and later time points, suggesting that these genes may have additional general roles during Hessian fly interactions with wheat that are not related to resistance or susceptibility at high temperatures. On the other hand, the upregulation of *Hfr-1* at higher temperatures by 1 DAH in the temperature-independent resistant lines alone suggests an involvement of this gene in an early defense strategy against the Hessian fly. While one of the temperature-independent resistant lines (PI 61862) also showed increased *Hfr-1* transcripts at 8 DAH at 30 °C, it may no longer be playing a role in defense as the larvae are already dead by that time point. *Hfr-1* encodes for a mannose-binding jacalin-like lectin [20]. Lectins are carbohydrate-binding proteins and well-documented to play a role in plant defense against pests [42, 43]. HFR-1 is shown to have antinutrient and antifeedant properties not only against Hessian fly [20] but also against the cereal grain aphid, *Sitobion avenae* [44]. Additionally, ingestion of the snowdrop lectin (*Galanthus nivalis* agglutinin) resulted in profound changes in the cytology of the Hessian fly larval midgut epithelial cells with massive disruption of brush border microvilli having deleterious effects on growth and development [45]. Similar disruptions of brush border microvilli are observed in the midguts of Hessian fly larvae feeding on resistant wheat as compared to well-developed microvilli observed in the virulent larvae feeding on susceptible wheat [22]. The larval antibiosis observed in the temperature-independent resistant wheat at 30 °C could be attributed to the binding of HFR-1 to the midgut epithelial cells and preventing the larvae from acquiring nutrients and starving them to death. Lack of feeding deterrents like HFR-1 in the heat-triggered susceptible durum wheat plants may allow the larvae to feed unhindered without any disruption to their midgut. Follow-up functional studies with HFR-1 are required for confirmation of its role in the temperature-independent resistant wheat lines. In the susceptible wheat (CItr 8637), *Hfr-1*, *Hfr-3* and *HfrDrd* were mostly downregulated at both temperatures in early as well as later time points, as observed previously in other Hessian fly-susceptible hexaploid wheat lines [19, 21, 40] confirming the suppression of defense responses by larval feeding, leading to establishment of plant susceptibility.

Rapid production of reactive oxygen species (ROS) radicals referred to as ‘oxidative burst’ is another well-documented adaptive defense strategy in plants to cope with insect herbivory [46–48]. An indication of occurrence of an oxidative burst in plants is the increase in levels of enzymes involved in both ROS production that leads to a hypersensitive response (programmed cell death) and ROS scavenging that limits the damage in response to the stressor [49, 50]. Although the wheat-Hessian fly interaction involves a gene-for-gene recognition, it does not resemble the classical interaction observed in plant-pathogen interactions [18, 40, 51]. ROS production is believed to be regulated by Class III peroxidases instead of the classical NADPH-dependent oxidase response in wheat [51, 52]. Our results showed that all durum wheat accessions (temperature-independent resistant, heat-triggered susceptible, and susceptible) exhibited increased transcript levels for ROS-associated genes in at least one or more time points without any specific expression pattern emerging for either of the temperature treatments (Fig. 7). Transcripts for *Pox* were up in one of the temperature-independent resistant wheat (PI 61862) and susceptible wheat at 20 °C but upregulated in the heat-triggered susceptible wheat (CItr 17647) and susceptible (CItr 8637) line at 30 °C. *Grx* and *Gst* transcripts were up-regulated in most lines in at least one time point when exposed to both temperatures. Interestingly, the oxidative burst pathway genes were also significantly upregulated in the susceptible wheat at 30 °C in response to Hessian fly larval feeding. Growing evidence suggests that ROS, besides potentially being responsible for cellular oxidative damage, can act as signal molecules during heat stress, leading to adaptive responses [53]. It is proposed that the heat-stress dependent ROS burst oxidizes the cellular environment, triggering redox-dependent signaling cascades [53]. In wheat, heat stress is better tolerated by plants that are subjected to heat priming by involving enhanced activity of antioxidant enzymes including peroxidases and glutathione reductase [54, 55]. Higher levels of expression of genes involved in oxidative stress resistance were observed in young *Arabidopsis* leaves exposed to higher temperatures suggesting their potential role in heat tolerance [56]. It is possible that the increased transcripts for the oxidative burst pathway genes observed at higher temperature could be an adaptive strategy to prime the plant for thermotolerance and prevent injuries to the cells. Larval feeding likely contributed to the increased transcript levels as differential expression of these genes was observed in the Hessian fly-infested tissue as compared to the uninfested controls in all wheat lines following heat stress.

Studies implicate the involvement of phytohormones during wheat heat stress and Hessian fly feeding [30]. OPDA (12-oxo-phytodieonic acid) was highly induced in the Hessian fly-resistant wheat Molly at 20 °C, but transiently decreased at 40 °C coinciding with resistance breaking down [30]. In a more recent study, the early application of phytohormones (salicylic acid, OPDA and auxin) enhanced wheat resistance to Hessian fly at 30 °C only if the larval density was low (1–2 larvae/plant) but not with higher larval density [31]. Our gene expression profile for *Opr3*, *Aoc* or *Aos*, however, did not reveal any specific expression pattern that could be correlated with either temperature-independent resistance or heat-triggered susceptibility to Hessian fly (Fig. 8). Like ROS radicals, JA has emerged as a key player in alleviating adverse effects caused by heat stress in plants [57]. In *Arabidopsis*, heat stress induced accumulation of jasmonates, including JA, OPDA, and JA-isoleucine, while plants lacking the JA signaling pathway displayed impaired heat tolerance [58]. However, in another study on heat stress in rice, genes involved in JA biosynthesis were suppressed with reduced JA levels [59]. These seemingly contradictory observations on the role of JA in heat response have been attributed to differences in the species, experimental conditions, or growth temperatures used in various studies [57]. Our gene expression results in tetraploid durum wheat are inconsistent with reports in hexaploid wheat warranting further studies at the metabolite level to quantify these phytohormones in the temperature-independent resistant and heat-triggered susceptible plants to ascertain their involvement in Hessian fly resistance in wheat.

The efficient utilization of wheat host nutrients following induced susceptibility is a prerequisite for the Hessian fly to successfully complete their development. This requires the induction of genes that are part of the amino acid and polyamine pathways as well as suppression of genes that play a part in cell wall biosynthesis. During compatible interactions (susceptible plants) the virulent larvae alter host metabolic pathways upregulating genes associated with susceptibility [8, 11–13] resulting in differentiation of a nutritive tissue at the feeding site that provides the developing larvae a diet rich in essential nutrients [7, 13–15] and making the plant stunted [60]. Following exposure to 30 °C, the heat-triggered susceptible (CItr 17647) line showed dramatically increased transcripts for the susceptibility-associated genes and amino-acid transporter genes resembling the biological process observed in the susceptible wheat, that were absent in the temperature-independent resistant durum lines (Fig. 5). Coinciding with the temporal increase in transcripts for genes associated with susceptibility and nutrients, the durum wheat accessions with heat-triggered susceptibility also exhibited a striking increase in cell wall permeability making the entire crown tissue a nutrient sink allowing nutrients to diffuse to the cell surface following Hessian fly infestation at elevated temperatures, resembling the susceptible wheat (Fig. 3). In contrast, at elevated temperatures, the wheat lines with temperature-independent resistance resembled the resistant wheat at 20 °C with limited NR staining and cell wall permeability. With increased cell wall permeability, we expected to see a decrease in transcripts of cell wall-associated genes in the heat-triggered susceptible wheat similar to the susceptible wheat (Fig. 6). Interestingly, the down-regulation of these cell wall-associated genes (*ß-glu*, *Exp* and *Ltp*) was delayed to 8 DAH possibly because it took longer for defense responses to turn off and a nutrient sink to develop in a plant that is resistant at 20 °C but is physiologically challenged at 30 °C with the larval attack. We also observed increased transcripts for the wall-associated gene encoding sucrose synthase (*SuSy*) in both the heat-triggered susceptible durum line as well as the susceptible wheat line at 30 °C possibly adding nutritive value for the larvae at the feeding sites by synthesizing sugars [9].

## Conclusion

In conclusion, we carried out in-depth studies on temperature-independent tetraploid durum wheat lines and demonstrated resilience at higher temperatures providing robust resistance against multiple Hessian fly biotypes. The molecular characterization of these lines identified *Hfr-1*, a mannose-binding jacalin-like lectin as possibly playing a role in maintaining resistance at higher temperatures. In contrast, the durum wheat line with heat-triggered susceptibility lost resistance at higher temperatures by up-regulating susceptibility-associated genes and mimicking a susceptible plant. Transcriptomics and metabolomics studies in the future with the temperature-independent resistant and heat-triggered susceptible durum wheat lines can provide further clues to modes and mechanisms of wheat resistance or susceptibility to Hessian fly when regulated by temperature. Additional glycomics profiling of heat-triggered susceptible wheat plants could reveal changes in plant cell wall structure and composition. The temperature-independent resistant durum wheat lines offer a valuable resource to wheat breeders and growers for providing Hessian fly resistance in geographical regions that experience elevated environmental temperatures affecting crop yield. Further characterization of the Hessian fly resistance gene in the temperature-independent resistant durum wheat would be valuable for wheat breeders to develop elite cultivars having the heat-resilience trait.

## Supplementary Information

Supplementary Material 1.

## Data Availability

Data is provided within the manuscript or supplementary information files.

## Abbreviations

ANOVA: Analysis of variance
C: Celsius
DAH: Days after egg-hatch
JA: Jasmonic acid
logFC: Log fold change
H: Hessian fly resistance gene
NR: Neutral red
OPDA: 12-Oxo-phytodieonic acid
qRT-PCR: Quantitative real-time reverse transcription polymerase chain reaction
ROS: Reactive oxygen species
SAS: Statistical analysis system
