# Supplementary material for: Genetic and phenotypic responses of temperature-independent Hessian fly-resistant durum wheat to larval attack during heat stress
Source: BMC Plant Biol. 2025 Feb 17;25:210. doi: 10.1186/s12870-025-06226-1 (PMC11831824; doi:10.1186/s12870-025-06226-1)
Supplement: Supplementary file 1 — Supplementary Material 1. [file 12870_2025_6226_MOESM1_ESM.docx]

**Table S1.** Primer sequences for gene expression analysis.

| **ID** | **Gene** | **Accession** | **Forward primer (5’-3’)** | **Reverse primer (5’-3’)** |
| --- | --- | --- | --- | --- |
| ***Endogenous Control*** | | | | |
| 18S | 18S ribosomal RNA | AY049040 | cggagagggagcctgagaa | tgcgcccggtattgttattt |
| ***Hessian fly-responsive defense-associated biomarker genes*** | | | | |
| *Hfr1* | Hessian fly-response gene-1 | AF483596 | cttaagacctctgctttctctaggtga | gatggtgatgcgctctaaacg |
| *Hfr3* | Hessian fly-responsive-3 | DQ462308 | gtccttgctgggctgatctc | tccggtcctaggccacagta |
| *HfrDrd* | Hessian fly-responsive disease resistance dirigent-like | JX501668 | acctcaaatccaggccacttct | gatctgacacccctgaagaggta |
| ***Hessian fly-responsive susceptibility-associated biomarker genes*** | | | | |
| *Mds1* | *Mayetiola* *destructor* susceptibility-1 | NM_001427967 | agtgcatgcgtttggacgta | tcgcacacacatcaaccaaat |
| *Aat* | Transmembrane amino acid transporter | BT009014 | tcggcgagctcaaggagtac | tgtgccgctgtgctactca |
| *Oat* | Ornithine aminotransferase | AF022915 | ggcacggaggcaaatgag | agtgaaataatgtcatgggaacca |
| *Odc* | Ornithine decarboxylase | KJ136117 | gctccaacttcaacggcttct | cgaatggcgtgtgctacgta |
| ***Cell wall-associated genes*** | | | | |
| *β-glu* | Beta-glucosidase | XM_037622760 | acggcgtgacaattggaaa | accttccggaacatcatagtaacc |
| *Exp* | Expansin | XM_037556028 | caaagggtgccatgcaatc | ccgctcgacgtggaagtt |
| *Ltp* | Lipid transfer protein | MK570866 | atgcaggtgcctcaagagtgt | cgcacttggagggaatgc |
| *SuSy* | Sucrose synthase | XM_037574605 | cagatactggagggcagattgtt | taagaaccatttcatcctccaatg |
| ***Oxidative stress pathway genes*** | | | | |
| *Pox* | Class III peroxidase | XM_037560167 | caggccgccttcttcga | tgccagtgagcacgttgatc |
| *Grx* | Glutaredoxin | XM_037622546 | ggagcgatctccggttcttc | caactcgcaaacaaagctaagc |
| *Gst* | Glutathione *S*-transferase | XM_037553886 | gtgccggtgctgatcca | ggcgaaagcctcgtcgat |
| ***Jasmonic acid pathway genes*** | | | | |
| *Aoc* | Allene oxide cyclase | XM_048680214 | ccaggtcaagctcaaccagatc | ggatgcccttgaggtagaaggt |
| *Aos* | Allene oxide synthase | XM_048715959 | tcgccggcaagtccaa | taggcgtcggcgatgaac |
| *Opr3* | 12-oxo-phytodienoate reductase | XM_037598243 | ggaaccacggatggtgaata | ggcaagtctggattggacag |
